# Development of a Knockout Competition in Basketball: A Study of the Spanish Copa del Rey

**DOI:** 10.3389/fpsyg.2019.02457

**Published:** 2019-11-08

**Authors:** Sergio José Ibáñez, Javier García-Rubio, David Rodríguez-Serrano, Sebastián Feu

**Affiliations:** ^1^Faculty of Sport Sciences, University of Extremadura, Cáceres, Spain; ^2^Facultad de Educación, Universidad Autónoma de Chile, Santiago de Chile, Chile; ^3^Faculty of Sports Sciences, University of Extremadura, Badajoz, Spain

**Keywords:** observational analysis, ball possession, style of play, game development, evaluation

## Abstract

Performance in basketball has been widely studied with regard to the results of the game using competition statistics. Few studies have analyzed the play process from a dynamic viewpoint regarding tactical actions and styles of play. The general objective of this research was to analyze the development in the styles of play of the teams participating in the Spanish Copa del Rey by studying the development of ball possessions (from start to finish) and the styles of play (attack and defense phases). The specific aim was to identify the relations between how possessions end and the style of play, as well as the relation between the duration of possession and the action of shooting and efficacy of the possession. All the matches corresponding to the Spanish Copa del Rey in basketball in the seasons 2015/2016, 2016/2017, and 2017/2018 were analyzed, comprising a total of 3,865 possessions. To this end, two groups of variables were characterized, which made it possible to define play, the development of possession (start and finish), and the style of play (attack and defense phase). An exploratory and descriptive analysis of the situational variables, development of possession, and style of play was carried out to characterize the competition. The Chi-squared test and Cramer’s V coefficient were used to estimate the association among the categorical variables, interpreting the association among the categories using contingency tables. The results show a greater number of attacks in the final stages of the matches, with short possessions that end in baskets or rebounds, and positional attacks and individual half-court defenses predominating. There were more shots in positional attacks and more fouls in transitions. It also was apparent that the competition is developing from 1 year to another. The Spanish Copa del Rey competition changes from season to season, revealing slight modifications in the teams’ styles of play, although there is stability in the fundamental play parameters, like the predominance of man-to-man defense, the duration of the attacks, and the use of screens. The style of play conditions the finalization of the possession.

## Introduction

The study of performance indicators is one of the emerging lines of research in the sports sciences, the purpose of which is to evaluate individual, group, or team performance (Hughes and Bartlett, 2002). The results of these investigations have great practical utility, as they help coaches to identify the key aspects of good or bad performance (Hodges and Franks, 2008). In order to obtain this information, coaches and researchers use video feedback and information technologies, in which observational methodology is fundamental (Liebermann and Franks, 2008).

Different tools have been used to obtain scientific evidence that makes it possible to analyze team performance in basketball. Video analysis/motion analysis is one of the most commonly used tools to evaluate team performance during a competition (Hughes and Bartlett, 2002). Video analysis permits the creation of notational recordings to objectively quantify performance indicators, in a valid and reliable manner (Nevill et al., 2008). It is also necessary for researchers to carry out the correct procedures for validating the collected data (O’Donoghue, 2014).

Research that analyzes performance indicators in basketball using observational methodology can be classified into those that analyze the product or final result of the game (static analysis) and those that analyze the play process or what occurs during the game (dynamic analysis) (Ibáñez et al., 2003; Fernandez et al., 2009), and some studies have implemented both approaches (Camerino et al., 2012). According to this, Gréhaigne and Godbout (2013) indicate that in literature reviews there are two strategies for measuring performance indicators. On the one hand, there are studies that analyze performance indicators from a static approach, concentrating on a determined moment, and on the others are the studies that use a dynamic approach, in which they analyze the development of play, bearing in mind how the play actions occur. Basketball is a complex, dynamic, and non-linear type sport. Thus, studies on the modeling of the game should record complex properties of the game under static (result description, quantify), dynamic (time, criticality), and self-organized (non-linearity, process description) complexity perspectives (Sampaio et al., 2013). In addition, basketball is considered as a stochastic process, were the internal logic of the game is defined according to game probabilities (Martínez-Santos et al., 2017).

The research that analyzes the product of the game is the static approach, and the results of the matches were studied. The data obtained tend to be of a quantitative type (game statistics), although sometimes qualitative and ordinal data are used. In this line of research, the studies focus on the analysis of the fundamental technical actions of basketball like shooting (Erčulj and Štrumbelj, 2015; Ibáñez et al., 2015) or the variables related with success in matches (Fierro, 2002; Ibáñez et al., 2008a,b; García et al., 2014), according to the specific playing position (Gómez et al., 2007), the number of players used in each team (Pojskić et al., 2009), or the type of competition (García et al., 2013).

The research that analyzes the process (dynamic approach) studies what happens during the development of the game. For that, the data can be obtained in real time or recorded. The type of data obtained is usually qualitative. The results of this line of research complement the limitations of static modeling, showing what occurs during play. Research on the process of play is directed at detecting individual, group, and team performance indicators. Studies on the play process for identifying individual performance indicators focus above all on the most relevant actions for the final results, the shots (Ibáñez et al., 2008a, 2009b; Martínez-Santos et al., 2017). Other studies aim at analyzing group tactical actions like inside play (Courel-Ibáñez et al., 2016) or on-ball screens (Gómez et al., 2015; Serna et al., 2017). Studies have also been performed using dynamic modeling that analyzes the actions of the whole team in the attack (Gómez et al., 2013) or defense (Gómez et al., 2010) phases.

Analyzing the competition using performance indicators, although useful and necessary, is not sufficient to explain all that happens (García-Rubio et al., 2015). This approach simplifies reality, omitting the context in which the actions occur without defining the behaviors or structures of the teams. In basketball, the team’s performance depends on the actions taken during the competition. For example, it has been shown that attacking from exterior and interior zones in the NBA increases the duration and effectiveness of the attacks (Courel-Ibáñez et al., 2016). Tactical actions have also been studied like screens (Lamas et al., 2015; Vaquera et al., 2016), attack (Škegro et al., 2011), or defense systems (Gómez et al., 2006), providing useful information for coaches and players.

It is not possible to generalize the results of investigations of performance indicators to the whole population practicing a sport, as the samples from which the data are collected come from competitions at a specific level of performance, with specific competitive formats and different sexes and age groups. To be able to compare the research results, it is necessary to study the different competitions as the players do not behave equally in all types of competitions (Ibáñez et al., 2009a). These results would have a dual purpose; on the one hand they could serve to provide a better general understanding of the corresponding sport, and on the other, they would increase knowledge of the specific sport in the studied context.

As well as carrying out comparative studies bearing in mind different situational variables, like the age and sex of the participants, their competitive level, and the competition type or format, it is also necessary to perform longitudinal studies to see the evolution of the game (Drust, 2010), to be able in this way to identify the stable performance indicators that make it possible to define a sport, and the changing ones that evolve along with the modification of the rules and the trends of the game. Basketball is constantly evolving (Ibáñez et al., 2018), something which is shown externally in the evolution of the performance indicators. Rule changes seek to increase the intensity of the competition, thus achieving more attractive and rapid play. Formal changes in the competition imply functional changes in play. The players have to assimilate these changes by adapting to the new rules, and to the new forms and functions that arise in the game. This implies changes in the players, both at a physical (Cormery et al., 2008) as well as technical (Pluta et al., 2014) and tactical levels (Štrumbelj et al., 2013). Some studies aim to identify the differences in the performance indicators between men and women (Sampaio et al., 2004), the evolution of the differences according to the players’ levels (García et al., 2010) or over several years in the same competition (Ibáñez et al., 2008a,b; Gómez et al., 2017). The majority of these studies have a quantitative and static approach, with the performance indicators or game statistics as the information source. Information is needed on the tactical behavior of the players and teams, that is, on the context of play and the interactions among the players (Courel-Ibáñez et al., 2016).

Play analysis requires the creation of different notation systems that make it possible for the coaches and researchers to record play actions (Hughes and Franks, 2004a). Research analyzing play in a dynamic manner needs specific variables to be determined to record the process of what went on in the match. Hughes and Franks (2004b) propose some generic variables that should be recorded in a basketball game, like a schematic representation of the basketball court, team, player, action, and time. Specifically, in basketball, for the analysis of the result of individual play, there are studies that propose the use of qualitative variables for studying shots like body gestures (technical action); defensive pressure; division of the court into microzones; the value of the shot; the player’s role, efficacy, previous action, period, quarter, etc. (Ibáñez et al., 2009a). For the study of group tactical actions, the researchers include other qualitative variables like offensive efficacy, division of the court into microzones, points scored, duration of possession, number of passes, location of pass, etc. (Courel-Ibáñez et al., 2016). To study team play, Gómez et al. (2013) propose the inclusion of variables like the number of passes used by each team during their ball possession, the number of players involved in ball possession, the defensive systems used by the defending team, the duration of the possession, and the use of screens. In all cases, the researchers must go through a process of design and validation of the system of categories (Ortega-Toro et al. (2019)), as well as evaluating the reliability of the observers when applying the designed instruments (Ibáñez et al., 2019). The diffusion of systematic observation systems makes it possible to understand a set of guidelines and procedures to carry out the observation and record the events. Moreover, other researchers can use the same observational instrument to confirm the validity of the data or obtain new evidence (More and Franks, 2004).

To the best of our knowledge, no studies have been found that show the evolution in team play in a dynamic fashion in a knockout competition. Thus, the general objective of the present study was to analyze the evolution in the way the teams participating in the Spanish Copa del Rey competition play, using a study of ball possession, analyzing the development of the possession (start and finish) and the style of play (attack and defense phases). The Spanish Copa del Rey is played as a knockout competition among the eight best-classified teams in the first stage of the regular ACB league (Spanish first division). The evolution in play was studied using an analysis of the last three editions. The characterization of the evolution of the game is complemented with two specific objectives: (1) to identify the relations between how possession ends and the style of play and (2) to analyze the relation between the duration of possession and the shooting action and efficacy of the possession.

## Method

### Research Design

An empirical investigation was performed using descriptive observational analysis (Ato et al., 2013), with an arbitrary observation code, in the habitual natural environment, in which the phenomenon was produced and in which the researcher did not intervene in what was being observed (Montero and León, 2007). In addition, due to the nature of the data, the research used a mixed methods approach (Anguera et al., 2014, 2017). Mixed methods is a new and emerging research approach in social sciences that combines statistical trends and stories to study human and social problems. Mixed methods used a combination of quantitative and qualitative data integrated in a research structure. Observational and descriptive methodologies have to converge in mixed methods (Anguera and Hernández-Mendo, 2014), not a mere juxtaposition of different data.

### Participants

The population selected for the sample for this study was the matches that were played as part of a knockout tournament for the Spanish Basketball Copa del Rey. This competition is played annually and is by single elimination, with the participation of the eight best clubs in the Spanish ACB League, the top official competition (**Appendix 1**). The sample of the study was composed of the 21 matches played during the last three seasons (seven matches per season). This research studied the way the teams played during the attack phase using an analysis of ball possession. For this study, ball possession was defined as the length of time that a team has the ball from when it gains control of it until it loses control of it, whether because of a violation, the loss of the ball or a shot at basket.

The total number of units for statistical analysis collected during these matches was 3,865 cases, corresponding to each possession of the teams participating in the competition (2016: *n* = 1,296; 2017: *n* = 1,297; 2018: *n* = 1,272). Table 1 shows the distribution of the possessions per quarter and period of the match.

**Table 1:** Distribution of possessions per quarter and period.

Season		Quarter					Period		
		First quarter	Second quarter	Third quarter	Fourth quarter	Overtimes	First half	Second half	Total
2015/2016	*n*	321	321	321	333	0	642	654	1,296
	%	24.8	24.8	24.8	25.7	0.0	49.5	50.5	
2016/2017	*n*	313	305	316	319	44	617	636	1,297
	%	24.1	23.5	24.4	24.6	3.4	47.6	49.0	
2017/2018	*n*	315	311	316	330	0	626	646	1,272
	%	24.8	24.4	24.8	25.9	0.0	49.2	50.8	
Total	*n*	949	937	953	982	44	1885	1936	3,865
	%	24.6	24.2	24.7	25.4	1.1	48.8	50.1	

### Variables

Three situational variables were selected as categories of observation (independent variables) in this study. These categories of observation defined how the possession of the ball was during the Spanish Copa del Rey. These categories of observation were the period (first, second, and overtimes); the quarter (first, second, third, fourth, and overtimes); and efficacy. Efficacy was understood to mean the number of points that the teams scored per ball possession. The value could vary between 0 and 4 points per possession.

To analyze the differences in the way of playing during the three seasons, two groups of criteria (dependent variables) were defined: development of possession and style of play.

In the development of the possession, the start was defined using three criteria: origin of the possession, role of the player who began the attack, and starting microzone. Furthermore, the end of the ball possession was defined with four new criteria: action that defined the end of the possession, shooting action, role of the player who ended the possession, and finishing microzone (Table 2).

**Table 2:** Criteria (dependent variables) that define the development of possession.

	Origin of possession (Gómez et al., 2009)	Player’s role	Microzone (Ibáñez et al., 2001b)	
Start of possession	Basket received	Guard	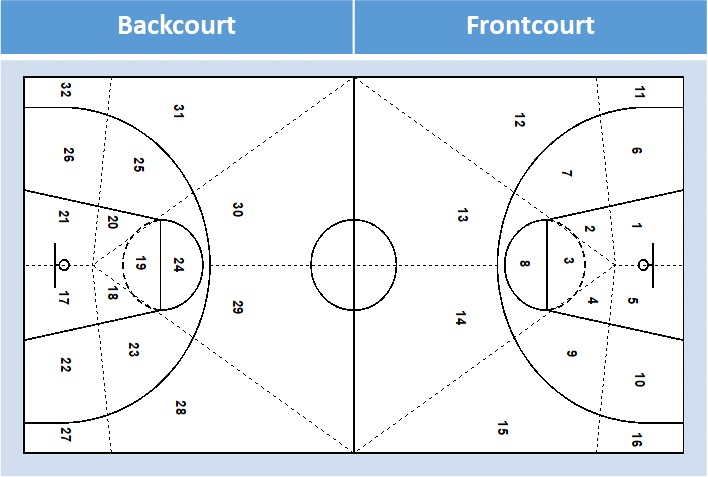	
	Free shot received	Forward		
	Defensive rebound	Centre		
	Offensive rebound			
	Back court baseline throw-in			
	Front court baseline throw-in			
	Back court sideline throw-in			
	Front court sideline throw-in			
	Steal			
	Midfield throw-in			
	Jump ball			
	**End of possession (Adapted from Gómez et al., 2009)**	**Shooting action**	**Player’s role**	**Microzone (Ibáñez et al., 2001b)**
End of possession	Shot	2 point converted	Guard	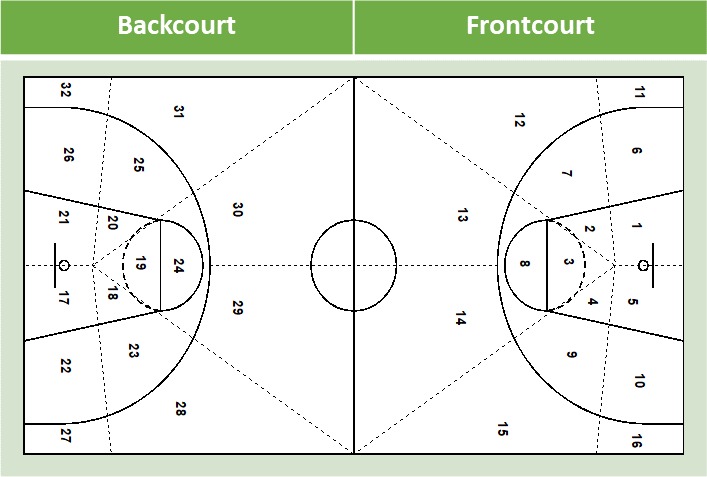
	Foul received	2 point failed	Forward	
	Offense foul/foul by team in control of the ball	3 point converted	Centre	
	Loss of ball	3 point failed		
	End of possession time (24 s)	Without shot		
	Jump ball			
	Traveling			
	Illegal Dribble			
	Technical foul			
	Deliberate Kick			

The style of play was identified using a criterion that defines the type of defense that the opposing team presented, and five criteria to define the style of play in the attacking phase: number of passes made, use of screens, number of participating players, type of attack, and duration of the possession (Table 3).

**Table 3:** Criteria (dependent variables) that define the style of play.

	**Type of defense (Gómez et al., 2009)**				
Defense phase	Individual half-court				
	Individual full court				
	Half-court zone				
	Full-court zone				
	Mixed				
	**Type of attack (Gómez et al., 2009)**	**Use of screens**	**Number of participants**	**Number of passes**	**Duration possession**
Attack phase	Positional	No screen	1–5	Number. 0, 1, 2, etc.	From 1″ to 24″
	Transition	Direct			
	Counterattack 1	Indirect			
	Counterattack 2	Both			

The qualitative variables of the study were defined using a categorical nucleus and its opening range or plasticity (Anguera and Hernández-Mendo, 2013, 2015).

### Procedure

The research was structured in several phases: (1) selection and design of the variables, (2) recording of the matches, (3) training of the coder, and (4) coding of the sample. In the first phase, the possession coding system was designed using a record sheet according to the criteria and categories (Tables 2, 3) designed “*expo facto.*” A group of expert coaches and graduates of Sports Sciences determined the selection of the variables. The previous studies by Ibáñez et al. (2001a,b,c) and Gómez et al. (2009) were taken into account to create the system of categories. In the second phase, the matches were downloaded from www.allsport-tv.com. Matches were watched with a video player, and coders were allowed to stop the game every time they need.

The coder was trained during the third phase, following the three stages proposed by Muñoz et al. (2018): the theoretical training stage, the practical training stage, and the supervised practice stage. Once the theoretical-practical training had finished, the actual training stage with the calculation of reliability and intra-observer concordance using Cohen’s Kappa for the qualitative variables and the Interclass Correlation Coefficient for the quantitative variables (O’Donoghue, 2013) were carried out.

To calculate reliability, two quarters, equivalent to 77 cases, were coded again at random. This number of cases is within the minimal range of 50 and 300 events (ball possessions) established for the calculation of reliability (González-Díaz and Iglesias-García, 2015). The time that elapsed between the first and the second viewing was 1 week. Intra-observer concordance in all the qualitative variables was almost perfect (Table 4; Landis and Koch, 1977). Similarly, the results showed high-level agreement among the quantitative variables (Table 5; Vincent and Weir, 2018). As the intra-coder concordance was optimal, all the matches were coded using the record sheet created *ex professo* and the collected data were then analyzed.

**Table 4:** Intra-observer concordance for qualitative variables.

	Development of possession							Style of play		
	Start of possession			End of possession				Defense phase	Attack phase	
	Origin of possession	Player’s role	Micro zone	End of possession	Shots	Player’s role	Micro zone	Type of defense	Type of attack	Use of screen
Cohen’s Kappa	1	0.878	0.914	1	0.983	0.898	0.957	0.916	1	0.869
Typical error	0.00	0.052	0.034	0.00	0.017	0.044	0.024	0.083	0.00	0.047
*n*	77	77	77	77	77	77	77	77	77	77

**Table 5:** Intra-observer concordance for quantitative variables.

	Style of play			
	Attack			
	Duration	Participants	Passes	Points
Interclass correlation coefficient	0.985	0.987	0.991	1
df	76	76	76	76
*n*	77	77	77	77

In the fourth stage, the sample matches were analyzed. All the competition matches were analyzed by an observer using systematic observation.

### Statistical Analysis

Firstly, a descriptive analysis was made of all the qualitative (*n* and %) and quantitative ($\bar{x}\;\mathrm{and}\;SD)$variables of the study. Chi-squared (*χ*^2^) and Cramer’s V (*V*_c_) were used to test the causal hypotheses, which make it possible to estimate the association among the categorical variables (Newell et al., 2014). These tests are appropriate because, for each level of the sample, it takes into account the differences between the recorded and the observed frequency. The data come from a multinomial variable (three championships of the Spanish Copa del Rey). The Chi-squared test is used for multinomial comparisons, using as a null hypothesis that all championship data have the same multinomial distribution. The interpretation of the degree of association among the variables was performed using adjusted standardized residuals (ASR) (>|1.96|) from the contingency tables (Field, 2013). The degree of association among the variables was estimated following Crewson’s proposal (2006), establishing four ranges of association: small (values < 0.100), low (values between 0.100 and 0.299), moderate (values between 0.300 and 0.499), and high (values > 0.500).

In this investigation, very low values were found in several categorical variables, so it was necessary to perform Fisher’s exact test using the Monte Carlo method to obtain exact results when the data did not comply with the underlying suppositions necessary to obtain reliable results with the use of the typical asymptotic method (Field, 2013).

K means clustering was used to specifically classify the quantitative variables of the study (number of passes, number of participating players, and duration of possessions) to be able to better identify the differences among the seasons. Thanks to this method, natural groups are identified within a large set of data that would not otherwise be evident (Sampaio et al., 2018). Before establishing the final groups in the manuscript, it was tested with different combinations of groups of the three variables. Finally, we decided, in our opinion of experts and different results, the final groups. Four groups were established to categorize the number of passes: group 1: low passing (zero and one pass, rebound and basket, rebound and long pass, rebound and counterattack, steal and attack, etc.); group 2: adequate passing (two, three, and four passes); group 3: moderate passing (five and six passes); and group 4: high passing (>seven passes). Three groups were defined for the number of participating players variable, with the result of the cluster as follows: group 1: low number of players (one or two players); group 2: moderate number of players (three or four players); group 3: teams (five players). Four groups were determined for the duration of possession: group 1 had a duration of 1–5 s (corresponding to the counterattacks or rapid attacks); group 2 included possessions of 6–11 s (attacks in transition); group 3 included possessions of between 12 and 17 s (organized positional attacks); and finally group 4 included possessions of between 18 and 24 s (very long attacks).

The cluster analysis allowed classifying all cases in these variables (seconds used in possession, players involved, and passes committed) into groups of similar characteristics instead of performing an analysis with all the possibilities. This decision was made because, from a practical point of view, as coaches we prefer to know the type of game according to the duration instead of the exact seconds. We classify plays in counterattacks, transition plays, position plays, and long plays, which are easier for coaches or conditioning coaches to understand. Other authors have grouped these variables in previous studies. Gómez et al. (2013) did this without clustering data.

Finally, a one-way ANOVA was performed to identify the differences among the quantitative variable (continuous) points scored over the three seasons. The software used was SPSS.24 (Armonk, NY: IBM Corp.).

## Results

The results of the one-way ANOVA in the variable points scored show the non-existence of differences in points per ball possession during the three seasons (*F* = 0.566, *p* = 0.568). The winning of points per ball possession did not vary in the three seasons analyzed. The mean number of points per ball possession during the 2015/2016 season was 0.86 ± 1.135, during the 2016/2017 season it was 0.88 ± 1.149, and during the 2017/2018 season 0.91 ± 1.140. The percentage of effective possessions is low, as in 59.8% of the possessions recorded no points were scored. The most common effective result was to score 2 points per possession (Table 6).

**Table 6:** Efficacy of possessions per season.

	2015/2016		2016/2017		2017/2018		Total	
	*n*	%	*n*	%	*n*	%	*n*	%
0 points	787	60.7	780	60.1	744	58.5	2,311	59.8
1 point	43	3.3	46	3.5	39	3.1	128	3.3
2 points	323	24.9	312	24.1	346	27.2	981	25.4
3 points	141	10.9	159	12.3	143	11.2	443	11.5
4 points	2	0.2	0	0	0	0	2	0.1
Total	1,296		1,297		1,272		3,865	

### Evolution of the Competition

Table 7 presents the differences found in the three seasons of the Spanish Copa del Rey analyzed in the different groups of variables.

**Table 7:** Results of the differences in the way of playing in the three seasons in the categorical variables.

	Variable	*χ*^2^	df	*p*	*f*	*p*	*V*_c_	*p*	Association level
S.V.	Period	88.154	4	**0.000**			0.107	0.000	Low
	Quarter	88.304	8	**0.000**			0.107	0.000	Low
Development of possession	Origin	26.949	20	0.137			0.084	0.137	
	Player’s role start of possession	25.884	4	**0.000**			0.058	0.000	Small
	Microzone start	146.297	52	**0.000**			0.138	0.000	Low
	End of possession				39.407	**0.000**	0.070	0.001	Small
	Shots	6.814	8	0.557			0.030	0.557	
	Player’s role end of possession	12.134	4	**0.016**			0.040	0.016	Small
	Microzone end				104.184	**0.000**	0.119	0.000	Low
Style of play	Use of screens	22.584	6	**0.001**			0.054	0.001	Small
	Type of attack	49.572	6	**0.000**			0.080	0.000	Small
	Type of defense				33.896	**0.000**	0.066	0.000	Small
	Possession duration cluster	11.049	6	0.087			0.038	0.087	
	Number of participants cluster	5.553	4	0.235			0.027	0.235	
	Number of passes cluster	18.006	6	**0.006**			0.048	0.006	Small

*S.V., situational variables. p < 0.001*.

To interpret the differences in the evolution of the game over the three seasons in the categorical variables, it is necessary to consult the contingency tables.

The results show a low relation between the period of the game and the quarter during the three seasons caused by the existence of possessions during the 2016/2017 season in an overtime with 44 ball possessions (ASR = 9.4).

### Development of Possession

The results of the variables that define the start of possession will be presented first. Table 8 shows the results of the distribution of possessions according to what originated it and the role of the player who began it. As can be seen, two variables stand out as the origin of a new possession. Obviously, they are baskets received or a defensive rebound. There are few significant differences among the seasons, as only in the 2016/2017 season were there more possessions than would be expected which began with a sideline throw-in from the front court, while during the 2016/2017 season there were less possessions starting with a sideline throw-in from the back court, and during the 2017/2018 season less possessions that started with a side line throw-in from the front court.

**Table 8:** Distribution of possessions according to the origin of the possession and player’s role.

		2015/2016			2016/2017			2017/2018		
		*n*	%	ASR	*n*	%	ASR	*n*	%	ASR
Origin of possession	Basket received	361	27.9	−1.4	381	29.4	0.1	391	31.0	1.4
	Free throw received	113	8.7	0.8	103	7.9	−0.5	103	8.1	−0.3
	Defensive rebound	298	23.0	−0.4	323	24.9	1.6	284	22	−1.1
	Offensive rebound	80	6.2	−0.8	78	6.0	−1.1	98	7.7	1.9
	Back court baseline throw-in	77	5.9	1.9	57	4.4	−1.1	57	4.5	−0.9
	Front court baseline throw-in	92	7.1	1.0	80	6.2	−0.4	77	6.1	−0.6
	Back court sideline rhrow-in	52	4.0	1.7	32	2.5	−2.1	44	3.5	0.4
	Front court sideline throw-in	103	7.9	−0.2	126	9.7	2.3	88	6.9	−2.1
	Steal	88	6.8	−0.3	82	6.3	−1.1	98	7.7	1.3
	Midfield sideline throw-in	25	1.9	−0.5	31	2.4	0.9	25	2.0	−0.4
	Jump ball	7	0.5	0.5	4	0.3	−1.0	7	0.6	0.5
Role	Guard	212	16.4	0.4	175	13.5	−3.0	232	18	2.6
	Forward	429	33.1	−3.7	517	39.9	2.4	492	39	1.3
	Centre	655	50.5	3.3	605	46.6	−0.1	548	43	−3.2

Differences have been found in the role of the players that start the possession and start the attacks. While in the 2015/2016 season there was a higher probability that the centers started the possession, in the 2016/2017 season it was the forwards, and in the 2017/2018 season it was the guards.

**Figure 1:**
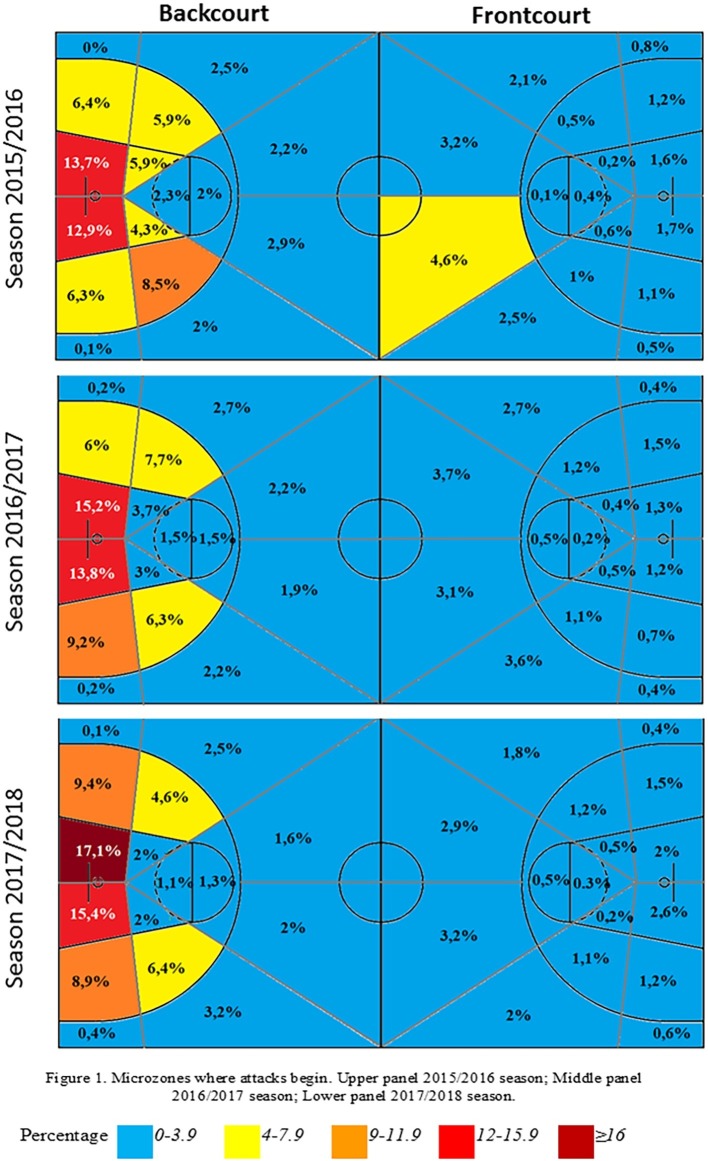
Microzones where attacks begin. Upper panel, 2015/2016 season; middle panel, 2016/2017 season; lower panel, 2017/2018 season.

Figure 1 shows the results of the start of the possession in the microzones of the court. The zones nearest to the defensive basket, zones 17 and 21, were the ones where possession mostly started, as it was where they received baseline throw-ins and got more defensive rebounds.

Differences were evident in the microzones where possessions began among the three seasons. During the 2015/2016 season, the zones where there was a greater incidence of possessions starting than would be expected were the zones near the free throw line, on the right, zones 18 (ASR = 3.1), 19 (ASR = 2.4), 20 (ASR = 4.5), and 23 (ASR = 2.4), as well as the right side of the offensive field, near the line in the center of the court, zone 14 (ASR = 2.2). The difference with regard to the 2016/2017 season is that competition possessions began differently in zones 15 (ASR = 4.5) the right side of the offensive field, and 25 (ASR = 3.0) the zone on the left near the free throw line in the defensive field. Finally, the differences with regard to the zones used for the beginning of the possession during the 2017/2018 season were zones 5 (ASR = 2.6), the zone near the opposing basket, 21 (ASR = 2.2) and 26 (ASR = 3.6), the left zone near the defensive baseline and 28 (ASR = 2.1), the side zone in the defensive field near the center court line.

The descriptive results of the end of possession are presented below. Table 9 shows the results of the distribution of possessions according to how they finished, the existence of shots and their efficacy, as well as the role of the player who ended the possession. It can be seen that in the three seasons analyzed, a shot at basket was the most common way for the possession to end, followed at quite a distance by personal fouls received and losses of the ball. It can also be seen that there is a higher percentage of two-point shots scored than three-point shots scored. Forwards are the ones who most tend to finish the possession and guards are those least likely to finish it, which may be due to the fact that there is only one guard per team on the field, in contrast to the forwards, where there are two.

**Table 9:** Distribution of possessions according to finish, shots, and role of the player who ends them.

		2015/2016			2016/2017			2017/2018		
		*n*	%	ASR	*n*	%	ASR	*n*	%	ASR
End of possession	Shot	788	60.8	−2.2	837	64.5	1.3	816	64.2	0.9
	Personal foul received	284	21.9	1.4	262	20.2	−0.5	252	19.8	−0.9
	Offensive foul	19	1.5	−0.7	17	1.3	−1.2	28	2.2	1.9
	Loss of ball	165	12.7	0.8	145	11.2	−1.3	158	12.4	0.4
	End of possession time	5	0.4	−0.9	10	0.8	1.4	6	0.5	−0.4
	Traveling	27	2.1	3.6	16	1.2	0.2	3	0.2	−3.8
	Held ball	5	0.4	1.4	1	0.1	−1.4	3	0.2	0.0
	Technical foul	1	0.1	1.4	0	0	−0.7	0	0	−0.7
	Deliberate kick	2	0.2	−1.9	9	0.7	1.7	6	0.5	0.2
Shots	2-point shot scored	273	21.1	−1.0	284	21.9	−0.1	292	23	1.0
	2-point shot missed	231	17.8	−0.8	248	19.1	0.7	236	18.6	0.1
	3-point shot scored	125	9.6	−1.0	144	11.1	1.1	130	10.2	−0.1
	3-point shot missed	233	18	−0.2	240	18.5	0.2	232	18.2	−0.1
	No shot	434	33.5	2.3	381	29.4	−1.5	382	30	−0.8
Role	Guard	321	24.8	1.7	315	24.3	1.2	258	20.3	−2.9
	Forward	565	43.6	−0.2	537	41.4	−2.2	593	46.6	2.4
	Centre	410	31.6	−1.3	445	34.3	1.2	421	33.1	0.1

Small differences can be identified in the distribution of the cases in these three variables. End of possession during the 2015/2016 season recorded a greater proportion of cases which ended with traveling (ASR = 3.6), with less possessions ending in a shot (ASR = −2.2). In contrast, during the 2017/2018 season, there were fewer violations for traveling (ASR = −3.8). These results coincide with those recorded in the variable type of shot, as during the 2015/2016 season there were more possessions ending without a shot (ASR = 2.3). Finally, the forwards increased their participation in finishing possessions during the 2017/2018 season with regard to other seasons (ASR = 2.4), with a lower participation in the previous season (ASR = −2.2). In contrast, the guards recorded less participation (ASR = −2.9).

**Figure 2:**
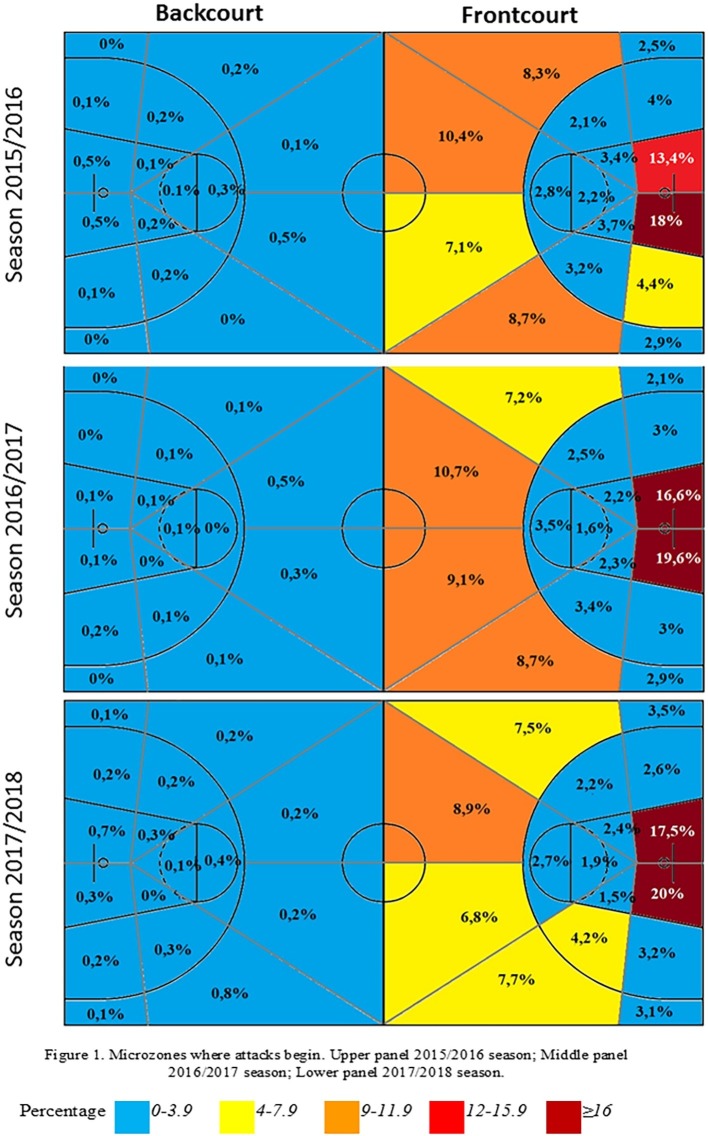
Microzones for ending possessions. Upper panel, 2015/2016 season; middle panel, 2016/2017 season; lower panel, 2017/2018 season.

Figure 2 shows the results of the distribution of possessions according to the microzone where they ended. It can be observed that the microzones where possessions ended in the three editions of the competition analyzed were 1 and 5, perhaps motivated by their position nearest the basket. It is easy to see the intentionality of the end of the possessions, commonly with a shot at basket. Firstly, the teams want to end the possession near the basket, to get greater efficacy. Secondly, the team opts to end the possession in zones which are far from the basket, outside the three-point line, as if they have to risk a basket shot, the score will be higher (3 points). Finally, in third place can be seen the tendency to use the intermediate zones to end possession.

There was a slight evolution in the microzones where possessions ended among the three seasons. During the 2015/2016 season, zones nearer the basket were used, zones 2 (ASR = 2.0), 4 (ASR = 3.4), and 10 (ASR = 2.0), while in the 2016/2017 season a far-off zone in front of the basket was used, zone 14 (ASR = 2.4), and in the 2017/2018 season a zone far from the basket but on the side was used, zone 11 (ASR = 2.1). These data show a tendency to use zones further from the basket to end possessions.

### Style of Play

Table 10 shows the results of the distribution of possessions according to the use of screens, the type of attack performed, and the type of defense that was encountered. It can be seen that in the majority of possessions there were no screens (62.3%), with the on-ball screen being the most commonly used (26.5%). With regard to the type of attack, in the three seasons the positional attack is clearly dominant (70.2%), followed by the transition (24.9%). To conclude, the most common type of defense used was by far the half court man-to-man defense (90.6%).

**Table 10:** Distribution of possessions according to the type of attack, use of screens, and type of defense.

		2015/2016			2016/2017			2017/2018			Total	
		*n*	%	ASR	*n*	%	ASR	*n*	%	ASR	*n*	%
Type of attack	Positional	828	63.9	−6.1	932	71.9	1.6	953	74.9	4.5	2,713	70.2
	Transition	404	31.2	6.4	301	23.2	−1.7	257	20.2	−4.7	962	24.9
	Counterattack first wave	47	3.6	−0.9	54	4.2	0.3	54	4.2	0.5	155	4.0
	Counterattack second wave	17	1.3	1.9	10	0.8	−0.6	8	0.6	1.3	35	0.9
Screens	No screen	816	63	0.6	805	62.1	−0.2	785	61.7	−0.5	2,406	62.3
	On-ball screen	319	24.6	−1.9	381	29.4	2.9	324	25.5	−1.0	1,024	26.5
	Off-ball screen	128	9.9	1.3	84	6.5	−4.0	138	10.8	2.7	350	9.1
	Both	33	2.5	1.0	27	2.1	−0.4	25	2	−0.7	85	2.2
Type of defense	Half court man-to-man	1,139	87.7	−4.1	1,180	91	0.6	1,182	92.9	3.5	3,501	90.6
	Full court man-to-man	129	10	4.4	80	6.2	−2.0	76	6	−2.3	285	7.4
	Half court zone	26	2	0.7	33	2.5	2.5	10	0.8	−3.3	69	1.8
	Full court zone	2	0.2	−0.7	3	0.2	0.0	4	0.3	0.7	9	0.2
	Mixed	0	0	−0.7	1	0.1	1.4	0	0	−0.7	1	0.0

An evolution can be observed in the style of play. The type of attack used by the teams evolved over the three seasons. While in the first season analyzed, 2015/2016, there was a greater proportion of transition attacks (ASR = 6.4), in the last season 2017/2018, the positional attack was predominant (ASR = 3.5). Specifically, in the attack phase, with regard to the use of screens there was also an evolution, as during the 2016/2017 season more on-ball screens were used (ASR = 2.9), but in the following season the off-ball screen was used more than expected (ASR = 2.7). Finally, the type of defense used also evolved slightly. In the 2015/2016 season defense was more aggressive, using a greater proportion of full court man-to-man defenses (ASR = 4.4), while the next season was more conservative, with the use of half court defenses (ASR = 2.5) changing to half court man-to-man defense during the 2017/2018 season (ASR = 3.5).

**Figure 3:**
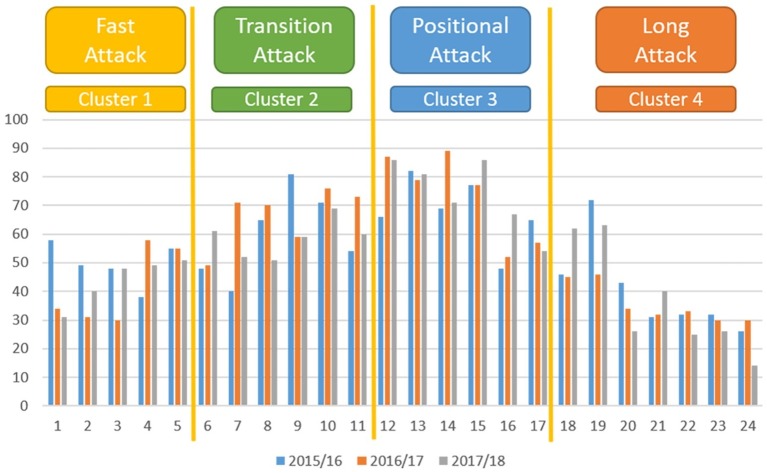
Duration of possessions.

Figure 3 shows the results of the distribution of possessions according to their duration. The mean duration of the possessions was 11.94 ± 6.24 s during the 2015/2016 season, 12.01 ± 5.89 s in the 2016/2017 season, and 11.98 ± 5.88 s in the 2017/2018 season. The predominance of the intermediate duration of possessions can be clearly seen, that is with attacks in cluster 3 with possessions of between 12 and 17 s (organized positional attacks).

No differences were identified in the number of players intervening in play over the three seasons. The most common styles of play in this type of competition are possessions in which the five players in the team intervene (62.4%), attacks with a lot of participation from the players, followed by possessions with a low participation of players (33.1%). Few possessions were recorded in which three or four players participated (4.6%).

In this type of competition, the number of passes per possession fluctuates between extremes. On the one hand, the most common possessions are those that involve few passes (59.1%) followed by those in which many players participate (26.8%). Either the play is rapid or involves very organized attacks (Table 11). There were significant differences in the evolution of the teams’ behaviors during this competition. In the 2015/2016 season, there were more possessions than expected in which a moderate number of players participated (ASR = 2.8), playing with a greater control of possession, while in the 2016/2017 season there was a higher percentage than expected of possessions with very few passes (ASR = 3.2), and play was quicker.

**Table 11:** Distribution of the number of passes.

	2015/2016			2016/2017			2017/2018			Total	
	*n*	%	ASR	*n*	%	ASR	*n*	%	ASR	*n*	%
Low passing (zero and one pass)	733	56.6	−2.3	813	62.7	3.2	739	58.1	−0.9	2,285	59.1
Adequate passing (two, three, and four passes)	45	3.5	1.6	35	2.7	−0.5	31	2.4	−1.1	111	2.9
Moderate passing (five and six passes)	171	13.2	2.8	121	9.3	−2.6	141	11.1	−0.2	433	11.2
High passing (>seven passes)	347	26.8	0.0	328	25.3	−1.5	361	28.4	1.5	1,036	26.8
Total	1,296			1,297			1,272				

### End of Possession

To complete our understanding of the style of play, a relation was identified between the variable role of the player who ended the possession and how the possession ended (*f* = 49.775; *p* < 0.000) with a small association (*V*_c_ = 0.081; *p* < 0.000). The forwards were the players who most intervened in the ending of possession (*n* = 1,695: 43.9%), followed by the centers (*n* = 1,276; 33%) and the guards (*n* = 894; 23.1%). There were differences among the three roles in the end of possession, as there was a higher number than expected of possessions where the guards lost the ball (ASR = 3.2), while the forwards violated the kicking rule (ASR = 2.7), and the centers were the ones that committed more attacking fouls (ASR = 3.7) and traveling violations (ASR = 2.1).

Table 12 shows the relations between how the possession ended and the style of play used during the Spanish Copa del Rey. The ending of possession is related to all the variables that define the style of play except the type of defense.

**Table 12:** Relations between the end of possession and the style of play.

	*χ*^2^	df	*p*	*f*	*p*	*V*_c_	*p*	Association level
Type of attack	107.400	24	**0.000**			0.167	0.000	Low
Use of screens				81.262	**0.000**	0.082	0.000	Small
Type of defense				56.629	0.362	0.038	0.891	Small
Possession duration cluster				155.000	**0.000**	0.115	0.000	Low
Number of participants cluster				108.018	**0.000**	0.118	0.000	Low
Number of passes cluster				144.963	**0.000**	0.116	0.000	Low

*S.V., situational variables. p < 0.001*.

The type of attack most commonly used in the Spanish Copa del Rey was the positional attack (70.2%) followed by the transition attack (24.9%). There was a higher number than expected of shots during the positional attacks (ASR = 6.9) and a lower number during transition attacks (ASR = −8.2). The opposite was true of the number of personal fouls received, which was lower than would be expected for positional attacks (ASR = −7.2), while in transition attacks there were more than expected (8.2). Lastly, there were more results than expected in which the possession ended with a violation of the traveling rule during the first wave counterattacks (ASR = 2.4). In summary, there were more shots in positional attacks, more fouls in transition, and more rule violations in first wave counterattacks.

Specifically, analyzing the use of screens during the attack phase and its relation to the end of possession, a small association has been found (*f* = 81.262, *p* < 0.000; *V*_c_ = 0.082; *p* < 0.000). The probability that the possession finalized with a shot was greater if on-ball screens were used (ASR = 6.1) than if there were no screens (ASR = −6.8). Similarly, the use of on-ball screens increased the possibility of ending the possession with a traveling violation (ASR = −2.3). On the contrary, when playing without screens, the possibility increased of ending the possession with a personal foul (ASR = 5.6) or loss of ball (ASR = 2.3). The use of screens during the attack phase favored ending with shots at basket, losing fewer balls.

The type of attack most commonly used in the Spanish Copa del Rey as a function of the duration of possession was the positional attack (33.4%), of between 12 and 17 s, and the long attack (18.7%), of between 18 and 24 s. The quick attacks (17.5%), of between 1 and 5 s, and the transition attack (20.4%), of between 6 and 11 s, were less commonly used in play. When the teams attacked quickly, they received more personal fouls (ASR = 6.2), but they lost more balls (ASR = 2.1) and did not finish with a shot (ASR = −7.1). The possibility of ending with a shot increased with transition attacks (ASR = 8.1) with fewer personal fouls (ASR = −8.2) and loss of balls (ASR = −2.4). Finally, long attacks did not guarantee a shot (ASR = −2.9), although they did guarantee a personal foul (ASR = 2.9).

A relation has been found between the number of players participating in the possession and the ending of the possession with a low association. The probabilities of shooting increased when there was a moderate number of players (three or four) (ASR = 4.7) or the whole team, five players (ASR = 6.5), and decreased when there was a low number of participating players (ASR = −8.7). In contrast, the number of personal fouls increased when the number of participating players was low, one or two players (ASR = 6.5), and was lower with a moderate number of players (ASR = −2.8) or the whole team (ASR = −5.1). The same was true of loss of balls, as they increased with the participation of a low number of players (ASR = 4.6), decreasing with the participation of a moderate number of players (ASR = −2.8) or the whole team (ASR = −3.2). The increase in players may cause the possession to end with a violation of the rules, like traveling (ASR = −2.2). Thus, during the Spanish Copa del Rey when playing as a team, there was a greater possibility of shooting at basket, although some rule violation may be committed, while quick play provoked personal fouls or loss of balls.

The style of play of the teams during the Spanish Copa del Rey as a function of the number of passes is associated with the way the possession ended. When the team used a high degree of passing (>seven), the probability of shooting decreased (ASR = −9.1), while if they used a low degree of passing (zero or one pass) (ASR = 3.2), an adequate degree of passing (two to four passes) or a moderate degree of passing (five or six passes) (ASR = 6.0), it increased. This association was inverted when the possession ended through a personal foul, increasing when there was a high passing rate (ASR = 6.9), with the probability decreasing in attacks carried out with fewer passes, low passing (ASR = −2.5), adequate passing (ASR = −2.4), or moderate passing (ASR = −4.6). Team play in which the whole team participated also increased the possibility of losing the ball (ASR = 4.2). The end of ball possessions was associated with individual play with an adequate number of players (two to four) (ASR = 4.5). The participation of a lower number of players facilitated shots at basket, while team play guaranteed the receiving of fouls.

### Duration of Possession

Table 13 shows the existence of relations between the cluster of duration of possession with the action of shooting and efficacy of possession.

**Table 13:** Relations between the possession duration cluster and shooting and efficacy.

Variable	*χ*^2^	df	*p*	*V*_c_	*p*	Association level
Shooting	158.611	12	**0.000**	0.117	0.000	Low
Efficacy	36.343	12	**0.000**	0.056	0.000	Low

*p < 0.001*.

When the teams played with quick attacks, of little duration, in 44.1% of the occasions there was no shot (ASR = 8.2) and 25.5% of the baskets scored were of two points (ASR = 2.4). When the length of possession increased, with transition attacks, there were more shots and only 19.7% of actions ended without a shot (ASR = −7.7). Similarly, an increase was evident in failed shots, as 24.9% of three-point shots (ASR = 5.4) and 21.8% of two-point shots (ASR = 2.7) were missed. When the teams played with organized positional attacks 28% of the actions ended without a shot (ASR = −2.8). Moreover, the proportion of possessions in which two-point shots were missed was greater than expected (ASR = 2.9). In the very long attacks, again there was a greater proportion than expected of no shots, that is 34.2% (ASR = 2.8). Very long attacks also produced a smaller proportion of cases of missed three-point shots (ASR = −2.1).

A relationship has also been identified between the duration of possession and its efficacy. Quick attacks involved a greater proportion of possessions which ended by scoring two-point shots (ASR = 3.8), and a smaller one than expected of those ending without scoring (ASR = −2.2) or by achieving three points (ASR = −2.8). When the teams used transition attacks, the proportion of possessions that ended in scoring three points increased (ASR = 2.0), decreasing the possibility of scoring one point (ASR = −2.3) or two points (ASR = −2.2). An increase in the duration of possession during organized positional attacks or long attacks did not imply a modification in the distribution of efficacy.

## Discussion

The general objective of this study was to analyze the evolution of the style of play in a knockout tournament, the Spanish Copa del Rey, by investigating ball possessions, comparing the development of possession and style of play. The characterization of the attacks in this competition makes it possible to identify relative stability in the style of play, indicating small differences in the development of possession and the style of play over the three seasons analyzed. Similarly, the relations identified between the ending of possession and the style of play suggest that when the teams play with positional attacks, they increase the possibility of making more shots, while when they play more quickly, in transition or first wave counterattack, they receive more fouls and commit more rule violations. The style of play is conditioned by the predominant defense, the half court man-to-man defense, provoking positional and transition attacks, using on-ball screens in 25% of possessions, with the participation of the five players in the team using many passes in the positional attacks or few in the transitions.

Many studies have analyzed sports competition as it relates to basketball over the years, but from a quantitative viewpoint (Ibáñez et al., 2008a,b; Puente et al., 2015), making it necessary to look for information on the play process. Studies like the present one contribute precise information for coaches and researchers on the play process in a dynamic manner. Previously, evolution in basketball was shown with studies of performance indicators and how they changed over time (Ibáñez et al., 2018), of the physical evolution of the players (Cormery et al., 2008; Štrumbelj et al., 2013) or their technical evolution (Mavridis et al., 2003). The studies that have analyzed longer time periods have shown greater differences and more stable tendencies than the present study. Coaches, players, and teams adapt their behavior according to the game context (Travassos et al., 2013). Thus, teams will modify their performance from 1 year’s competition to the next according to the performance of all the teams in the previous edition. As this study has shown, basketball evolves slowly, year after year, until it shows different styles of play which represent different eras.

It has been shown that teams are more effective when they do not use passes or when more than four players intervene at the end of matches (Gómez et al., 2013). When no pass is used, a study of play suggests a ball recovery action and rapid advantageous finish, whether with an offensive rebound or midcourt steal. The participation of four or more players in a possession indicates group play to create a space in the time needed to score (Mavridis et al., 2003; Gómez et al., 2013), where an effort by all the team is needed to generate this space in the most effective zone in this and other studies, the restricted zone/3 s, (Gómez et al., 2013). In the study of the Spanish Copa del Rey, it has emerged that the teams seek efficacy using two play strategies, scoring a basket quickly or looking for quick attacks that guarantee a shot.

The results show that quick attacks cause an increase in the proportion of cases in which two points are scored. An increase in the duration of possession does not imply an increase in the number of points that can be scored. In this study, the mean duration of the attacks was around 12 s in the three seasons. This length of time is very similar to that found in the competitions in the regular league, like the professional Spanish basketball leagues, ACB (12.47 s) and LF (11.82 s) (Romarís et al., 2016). In winning teams, an increase in the rhythm of play translates into a higher number of balls recovered and two-point shots scored. While in losing teams, an increase in the rhythm of play leads to an increase in fouls committed (Sampaio et al., 2010). Shorter attacks, in this study, increased the number of two-point shots scored. This is why teams seek to increase the number of attacks in the form of counterattacks and transition attacks.

The present study presents a percentage of transition attacks/counterattacks of about 25%, while in previous competitions the percentages were lower, both in national team competitions (13%) (Škegro et al., 2011), European club competitions (15.05%), and the NBA (20.2%) (Selmanović et al., 2015). Transition/counterattacking play is the one that generates more efficacy (Tsamourtzis et al., 2005; Gómez et al., 2009). Transitions are more unpredictable than organized attacks due to their spontaneity. To prepare them, a defense must be focused on what generates losses or defensive rebounds (Selmanović et al., 2015), as these are the actions that lead to this type of situation. In fact, in the present study the transition attacks ended in a foul, while the positional attacks ended in shots. Teams recognize the efficacy of this type of attack, avoiding the success of the more dangerous players with personal fouls which stop play (Gómez et al., 2016b), allowing the defenses to reorganize in order to be more effective. Thus, players can be disqualified for committing their fifth personal foul. This is a critical moment in the match which usually occurs at the end (Gómez et al., 2016a), when the players are more vulnerable to psychological performance issues due to the pressure of time. Furthermore, players and coaches have to bear in mind the number of fouls that each player on the field can commit when stopping counterattacks with fouls, and the possibility or not of free throws for the opposing team, or the match time remaining, bearing in mind that if a fifth personal foul is committed by a player, this will have negative effects for his or her team and positive effects for the opposing team (Gómez et al., 2016a,b).

Positional attacks, on the other hand, represent the majority of attacks, showing they are of great importance for team preparation (Milanović et al., 2014). Basketball teams have to be prepared to deploy many alternatives in static play, all types of play without screens and with direct or indirect screens. The duration of the attacks in the seasons was around 12 s, with an average of three players and two passes in relation to previous studies (Fernandes and Tavares, 2002; Gómez et al., 2010). Duration of possession and the increase in the number of passes is reflected in a higher number of assists and shots from near the basket (Gómez et al., 2006). One of the characteristics of winning teams is that they are able to adapt the duration, increasing the number of passes and decreasing the number of bounces depending on the different defensive systems proposed by the rival teams (Stavropoulos and Foundalis, 2005).

In elite European basketball competitions, the use of on-ball screens has become one of the main movements in current basketball (Marmarinos et al., 2016) and is used about 35% of the time in the Olympic Games (Lamas et al., 2011) or 41% of the time in the EuroLeague (Marmarinos et al., 2016), representing one of the most important actions for ending during attack systems (Vaquera et al., 2016). In this study, the presence of the on-ball screen in 26.5% of the cases in the three seasons was a lower value than that found in previous studies. In fact, the best teams should prepare different options for attack and defense to obtain success (Trninić et al., 2002). The on-ball screen is used to obtain an advantage through a mismatch, both because of the speed of the guards to dribble to a larger and heavier defender, or by the screener continuing toward the basket and, even, continuing with an opening for a long throw (Mattheos et al., 2010). To play effectively, it is necessary to study the players’ behaviors in these situations, especially when they are able to generate advantages for the defenders. When they can understand this part of play, coaches will be able to design better and more effective training situations for improved preparation (Gómez et al., 2015).

The majority of defenses used in the seasons analyzed were man-to-man defenses (Mexas et al., 2005; Gómez et al., 2010). This defensive system is based on maintaining the ball far from the basket, with the individual responsibility of each defender with his or her counterpart and group responsibility for the counterparts of the teammates. This defensive system, among other advantages, makes it possible to decrease the opponent’s opportunities for counterattacking (Gómez et al., 2010), as the defenders do not go directly to their zones of responsibility for zone defense. Furthermore, zone defenses permit more time for carrying out a throw from far away (Serna and Muñoz, 2015), as the defenders do not have an assigned attacker, just a zone. At this level of performance, as shown by our results, zone defenses are used only marginally, to change the rhythm of play of the attackers and generate surprise and uncertainty (Gómez et al., 2010; Ortega et al., 2010).

## Conclusions

The Spanish Copa del Rey competition changes from season to season, showing small modifications in the teams’ styles of play, although a certain stability has been identified in the fundamental play parameters, like the predominance of man-to-man defense, the duration of attacks, or the use of screens. The style of play conditions the ending of possessions. The relationship between the duration of the possessions and their efficacy shows that the teams must change rapidly from a defensive to an offensive role to seek to end the possessions quickly, as an increase in possession duration does not necessarily imply increasing the number of points scored. When the teams cannot end their attacks quickly, coaches should design positional attacks in which the greatest number of team players participate, using screens which facilitate shots at basket.

The analysis of play action from a qualitative point of view is useful both for coaches and players. To thoroughly understand a competition of this type will permit coaches to better prepare their players and teams. In this competition, the attacks are short, with very direct systems where two or three players touch the ball. Defense coaches should plan their defenses according to the type of attack. The players, for example, should be more intense in the first half of the possessions, making it difficult for the attackers to pass to the key player in each system (an average of three passes per possession), forcing them to take the ball to another zone of the court, modifying the options for attack and delaying the finish. Another option for modifying the attackers’ rhythm of play, and knowing that about half the time the center starts the possession, is for the center’s defender to make it difficult to receive the ball after the start of the possession.

## Data Availability Statement

The datasets generated for this study are available on request to the corresponding author.

## Author Contributions

SI contributed to the conceptualization, data collection, formal analysis, investigation, methodology and software development, visualization of the data, and writing the original draft. JG-R contributed to the supervision, writing the original draft and review, and editing the manuscript. DR-S contributed to data collection and to writing and editing the manuscript. SF contributed to funding acquisition, supervision, writing review, and editing the manuscript.

### Conflict of Interest

The authors declare that the research was conducted in the absence of any commercial or financial relationships that could be construed as a potential conflict of interest.

## Appendix 1 Teams participants in ACB Copa Del Rey in each analyzed season

**Table:**

Season 1	Season 2	Season 3
Iberostar Tenerife	Valencia Basket	Iberostar Tenerife
Baskonia	Iberostar Tenerife	Unicaja Málaga
Real Madrid	Real Madrid	Barça Lassa
Morabanc Andorra	Unicaja Málaga	Valencia Basket
Valencia Basket	Montakit Fuenlabrada	Kirolbet Baskonia
Herbalife Gran Canaria	Herbalife Gran Canaria	Divina Seguros Joventut
Barcelona Lassa	FC Barcelona Lassa	Real Madrid
Unicaja	Baskonia	Movistar Estudiantes
